# Roadmap Guided Direct Percutaneous Vertebral Artery Puncture for Mechanical Thrombectomy of Acute Basilar Artery Occlusion: A Technical Case Report and Review of the Literature

**DOI:** 10.3389/fneur.2021.789347

**Published:** 2022-01-06

**Authors:** Jawed Nawabi, Georg Bohner, Eberhard Siebert

**Affiliations:** ^1^Institute of Radiology, Charité - Universitätsmedizin Berlin, Humboldt-Universität zu Berlin, Freie Universität Berlin, Berlin, Germany; ^2^Berlin Institute of Health (BIH), BIH Biomedical Innovation Academy, Berlin, Germany; ^3^Institute of Neuroradiology, Charité - Universitätsmedizin Berlin, Humboldt-Universität zu Berlin, Freie Universität Berlin, Berlin, Germany

**Keywords:** mechanical thrombectomy (MT), direct vertebral puncture, stroke, large vessel occlusion, bail out

## Abstract

Access techniques for mechanical thrombectomy normally include percutaneous puncture of the common femoral or, more recently, the radial artery. Although target vessel catheterization may frequently not be devoid of difficulties *via* both routes, the vast majority of mechanical thrombectomy (MT) cases can be successfully managed. However, in a significant minority of cases, a stable target vessel access cannot be reached resulting in futile recanalization procedures and detrimental outcomes for the patients. As such, in analogy to direct carotid puncture for anterior circulation MT, direct vertebral artery (VA) puncture (DVP) is a direct cervical approach, which can constitute the only feasible access to the posterior circulation in highly selected cases. So far, due to the rarity of DVP, only anecdotal evidence from isolated case reports is available and this approach raises concerns with regard to safety issues, feasibility, and technical realization. We present a case in which bail-out access to the posterior circulation was successfully obtained through a roadmap-guided lateral direct puncture of the V2 segment of the cervical VA and give an overview of technical nuances of published DVP approaches for posterior circulation MT.

## Introduction

Access techniques for mechanical thrombectomy normally include percutaneous puncture of the common femoral or, more recently, the radial artery. Although target vessel catheterization may frequently not be devoid of difficulties *via* both routes, the vast majority of mechanical thrombectomy (MT) cases can be successfully managed. However, in a significant minority of cases, a stable target vessel access cannot be reached resulting in futile recanalization procedures and detrimental outcomes for the patients (1). As such, in analogy to direct carotid puncture for anterior circulation MT, direct vertebral artery (VA) puncture (DVP) is a direct cervical approach, which can constitute the only feasible access to the posterior circulation in highly selected cases. So far, due to the rarity of DVP, only anecdotal evidence from isolated case reports is available and this approach raises concerns with regard to safety issues, feasibility, and technical realization. We present a case in which bail-out access to the posterior circulation was successfully obtained through a roadmap-guided lateral direct puncture of the V2 segment of the cervical VA, and give an overview of technical nuances of published DVP approaches for posterior circulation MT.

## Case Presentation

A 70-year-old female patient presented with a right-sided hemisyndrome and an NIHSS of 4 for 6 h in the emergency department in the morning. Clinical symptoms improved spontaneously before critically deteriorating suddenly at 7 p.m. the same day to a GCS of 6.

## Imaging

**Figure 1:**
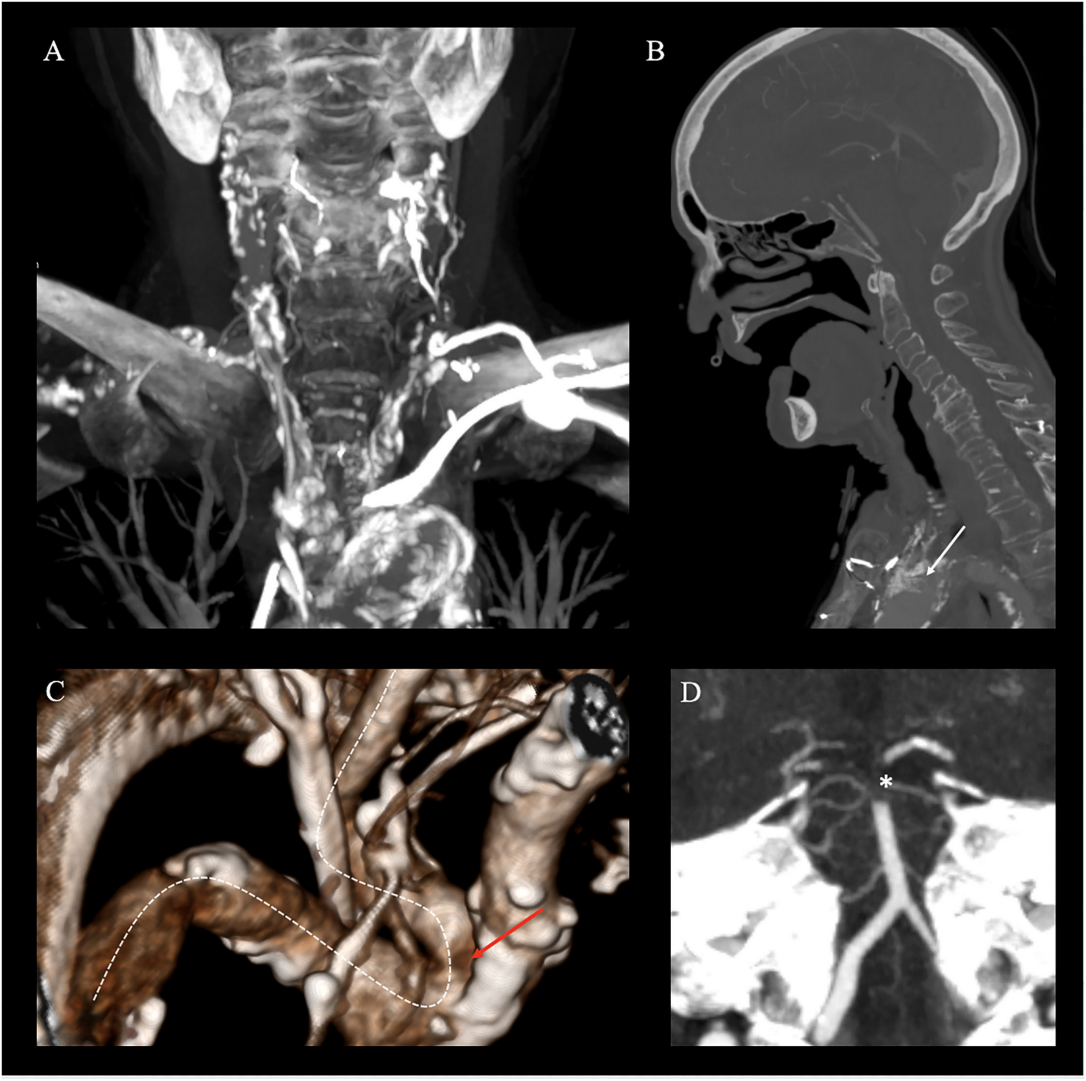
Non-invasive emergency imaging. Emergency imaging of a 70-year-old female patient with acute basilary artery occlusion (BAO), on. **(A–D)** Computed Tomography Angiography (CTA) in MIP reconstruction shows severe atherosclerosis of the aorta and cervical arteries (coronal plane), **(A)** a prolapsing stent of the common carotid artery into the aortic arch (white arrow, sagittal plane), **(B)** kinking and stenosis of the right subclavian artery (white dotted line) and an osteal right VA stenosis on the right (red arrow) with a steep-angled take-off from the subclavian artery (white dotted line, 3D reconstruction), **(C)** and distal occlusion of the basilary artery extending to both P1 segments of the posterior cerebral artery [*; white star, paracoronal plane, **(D)**].

Imaging upon admission identified neither signs of acute ischemic stroke nor a detectable vessel occlusion. Imaging upon sudden clinical deterioration demonstrated early ischemic changes in the posterior cerebral artery (PCA) territories bilaterally and a basilar hyperdense artery sign. CT angiography (CTA) showed a distal basilar artery (BAO) occlusion, extending from the basilar tip into both proximal segments of the posterior cerebral arteries (PCA, P1 segment; Figure 1D). Regarding vascular access, a type three aortic arch, severe atherosclerosis of the supra-aortic vessels (Figure 1A), an occluded stent in the proximal common carotid artery (CCA) prolapsing into the aortic arch (Figure 1B), a direct take-off of the left severely long segmentally stenotic VA from the aortic arch immediately adjacent to the stent, and an osteal VA stenosis on the right with a steep-angled take-off from the subclavian artery were noted (Figure 1C).

## Treatment

**Figure 2:**
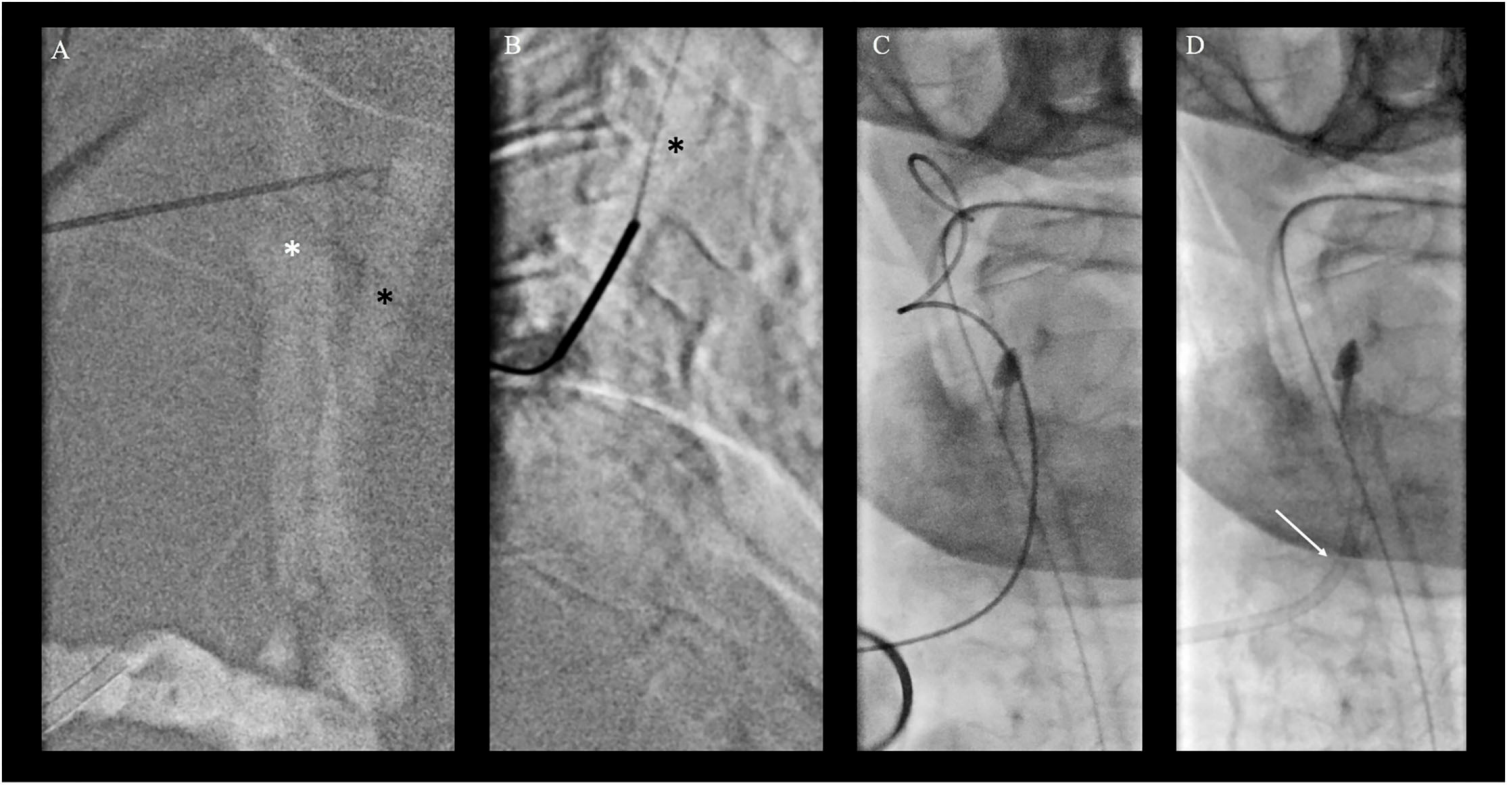
Bailout direct cervical vertebral artery (VA) access. **(A)** Roadmap injection through the right radial artery shows right common carotid artery (*; white star) and dorsally located VA of the V2 segment (black arrow) in posterior-anterior projection with a puncture needle for better orientation. **(B)** Direct puncture of the V2 segment of the VA under roadmap guidance in lateral projection at the level of C4/5 of the cervical spine. The micropuncture needle was percutaneously advanced from the far lateral cervical triangle at the height of C5 puncturing the lateral aspect of the V2 vertebral artery at the C4/5 level. **(C, D)** Placement of a microsheath via the microwire **(C)** and subsequent exchange for a standard 10 cm 5F femoral sheath over a standard 0.038 steel exchange wire **(D)**.

**Figure 3:**
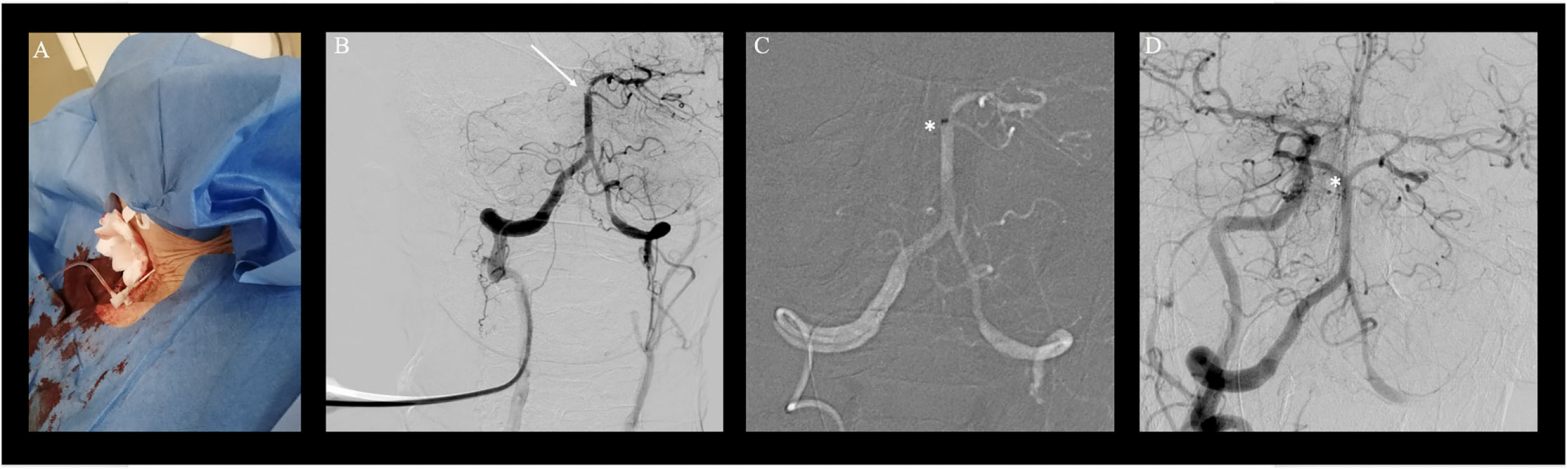
Transvertebral mechanical thrombectomy for basilary artery occlusion (BAO). Transvertebral mechanical thrombectomy for basilary artery occlusion (BAO). **(A)** Access site of the patient with a femoral sheath in place after single wall percutaneous puncture of the right vertebral artery of the V2 segment at the level of C4/C5 of the cervical spine. **(B)** Transvertebral injection and documentation of persistent BAO and right posterior cerebral artery occlusion (white arrow). **(C)** Wireless navigation of the large bore aspiration catheter (SOFIA) up to the occlusion site (*; white star). **(D)** Control fluoroscopy via right brachial artery with full recanalization of the occlusion site (white star).

Clinical indication for treatment was based upon a patient's decree explicitly stating maximal therapy and according to the early time window considering the second event, treatment was pursued despite the patient's compromised clinical state. As intravenous thrombolysis was contraindicated due to neoplastic disease, the intubated patient was directly transported to the angiosuite for MT. Both groins were pulseless. Since the right femoral artery was knowingly occluded, a nine French sheath was placed in the nearly pulseless left common femoral artery (CFA) for access under ultrasound guidance. Despite the passage of tight stenosis in the left common iliac artery, catheterization of both vertebral arteries was unsuccessful during multiple attempts using various select catheters and wires. Thus, a right transradial approach to gain access to the right dominant VA with a six French radial sheath was undertaken. However, multiple selection attempts of the right VA remained futile due to the kinking of the middle segment of the subclavian artery as well as the steep-angled VA take-off and the ostial stenosis (Figure 1). As a bail-out access procedure, a direct percutaneous lateral cervical VA access was entertained considering the acute nature with a short time interval from clinical deterioration to groin puncture and overall dismal prognosis of persistent BAO. As in total 60 min had elapsed (30 min for femoral access and 30 min for radial access), the direct puncture was favored over a more time-consuming surgical cutdown. Under road mapping, *via* the right subclavian catheter injection, a micropuncture needle was percutaneously advanced from the far lateral cervical triangle at the height of C5 puncturing the lateral aspect of the C2 vertebral artery (Figures 2A,B). Following the return of arterialized blood, the microsheath was laced *via* the microwire and subsequently exchanged for a standard 10 cm 5F femoral sheath over a standard.038 steel exchange wire (Figures 2C,D). The operating access area for anatomical orientation of the V2 access is shown in Figure 3A. Persistent occlusion of the basilar tip and the right PCA (Figure 3B) was confirmed by direct vertebral sheath injection (Figure 3B). A five-French aspiration catheter (SOFIA) was wirelessly advanced up to the basilar embolus and a single aspiration maneuver (wADAPT technique) was performed (Figure 3C). The aspiration catheter remained obstructed by the thrombus and was removed under manual constant suction with combined parallel suction on the sheath, which itself became blocked upon removing the aspiration catheter. Control imaging *via* the subclavian catheter showed complete recanalization of the basilar tip and the PCA territories (TICI3; Figure 3D). The V2 segment demonstrated a contrast filling defect at the tip of the sheath representing residual thrombus. Despite prolonged and vigorous aspiration on the sheath it stayed blocked and no more thrombus could be retrieved. Thus, the sheath was removed under aspiration and manual compression was applied to the puncture site for 15 min. Control imaging *via* the subclavian catheter after manual compression as well as follow-up CTA after 24 h demonstrated no extravasation or pseudoaneurysm, persistent TICI 3 recanalization, and a residual wall adherent thrombus at the puncture site not impeding antegrade flow.

## Follow-Up Imaging and Clinical Outcome

**Figure 4:**
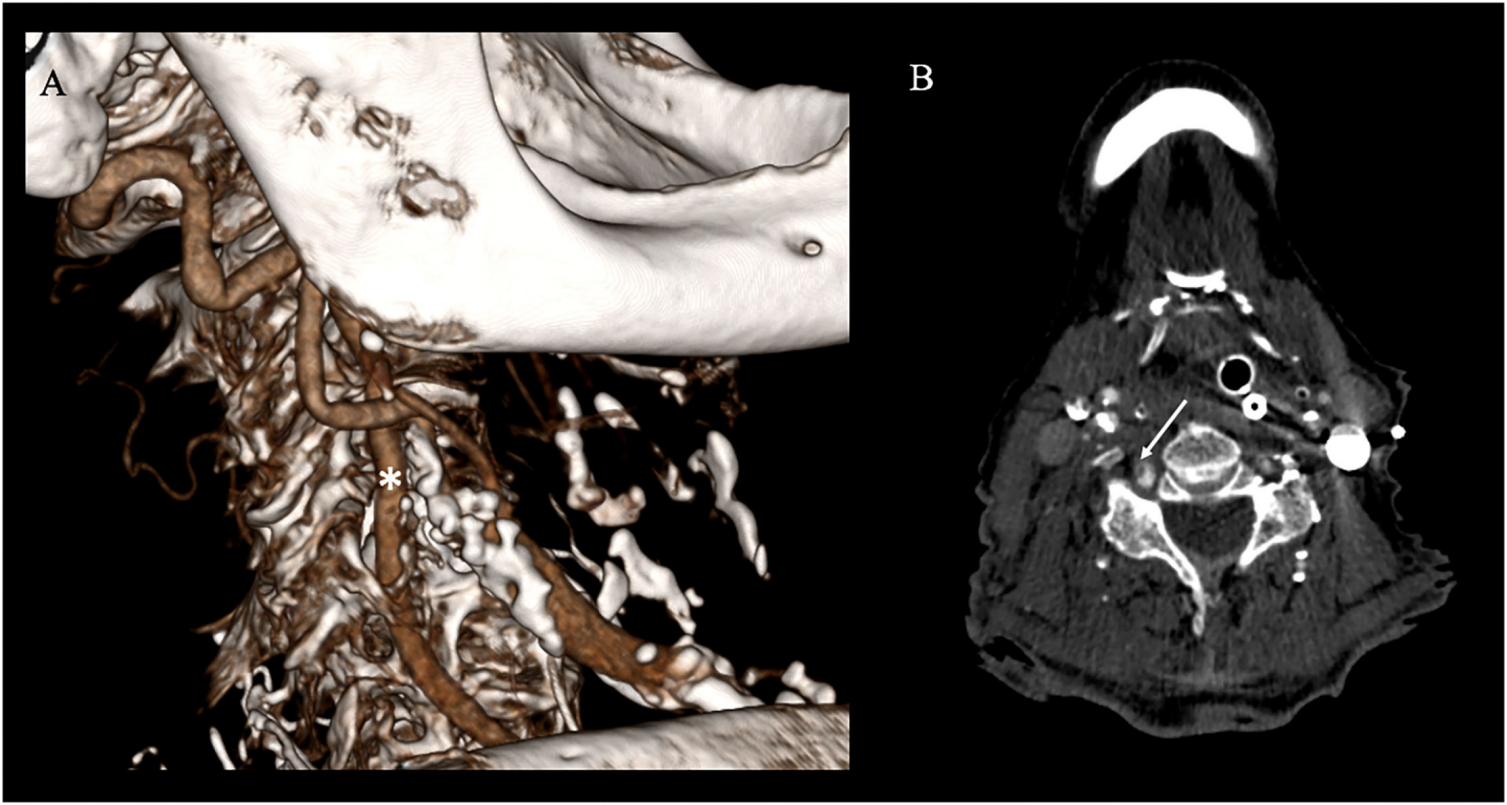
Follow-up vascular imaging. **(A)** CT-angiography (CTA) with sagittal 3D-reconstruction shows continuous contrast filling of the vertebral artery (*; white star) and no vascular. **(B)** However axial CTA shows evidence of a residual wall adherent thrombus at the former access site section but no cervical hematoma (white arrow).

The follow-up imaging done for 24 h has demonstrated extensive territorial infarctions in the right occipital lobe and pons, while CTA confirmed persistent complete recanalization, stable wall-adherent thrombus at the VA puncture site, and absence of a local dissection, pseudoaneurysm, or hematoma (Figure 4). Given the overall poor prognosis medical treatment was stopped in joint determination with the patient's family members and the patient expired.

## Discussion

Acute BAO accounts for about 1–4% of all acute ischemic strokes (AIS) with severe disability and mortality rates ranging up to 86% (6). Substantial clinical evidence supports the clinical benefit of MT, which is currently considered the standard of care by most (7–10), althoughevidence from randomized-controlled trials (RCT) is lacking (11). According to the literature, a significant minority of interventions are unsuccessful in terms of vessel recanalization of (20%) and of this 17% due to the inability to reach the occlusion site (12). For these cases, rescue vascular access techniques, such as early direct carotid or vertebral puncture or surgical arterial access, should be considered on an individual basis (1).

According to the current standard of treatment, vascular access for MT is usually achieved by a femoral arterial puncture for catheterization of the supra-aortic vessels. Yet, severe vessel and aortic arch remodeling secondary to chronic hypertensive disease may hinder timely and successful selective catheterization of the target cervical vessel. Recently, evidence points to adopt a radial-first strategy for both diagnostic procedures and MT were increased, especially for BAO (13, 14). Yet, radial-first attempts may also remain unsuccessful in a small but important proportion of cases and some anatomies are likely to be problematic for both approaches (15, 16). In these highly selected cases, DVP may provide an elegant solution to quickly establish vascular access and provide a stable platform with the excellent transmission of torque and forces in relative proximity of the target vessel occlusion for subsequent MT of BAO.

Direct VA puncture (DVP) has been performed under fluoroscopy, US and as of recently also under road mapping (2, 3). Percutaneous angiography of the vertebral artery was first described in 1950 by Lindgren (17) who stated that the examiner presses two fingers of the left hand in between the brachiocephalic vessels and the aerodigestive lumen, at the level of C4. A catheter needle is then introduced toward the midline until it touches the vertebral body and directed upward and outward until it glides between adjacent transverse processes to hit the vertebral artery. Lindgren's technique was further refined for diagnostic angiographies by Sjögren in 1953 and adapted in a similar approach during endovascular treatment of basilar tip aneurysms by Weill and Cognard et al. in 1998 (17). Over time, a variety of puncture sites and guidance strategies for DVP access have been described and were recently reviewed by Elhorany et al. (2). Their results underline that clinical evidence supporting different techniques of DVP for MT in the posterior circulation is extremely limited. Only four cases other than this one were reported in the context of MT for BAO, three describing access to the V3 segment (using ultrasound guidance or road mapping) and only one describing V2 access using ultrasound (2–5). Their image [Figure 3, (7)] these operators seem to have used a far lateral approach, too, although the puncture site has not been documented on the fluoroscopy image. While limited previous reports of endovascular access by direct percutaneous V2 puncture exist, they have only been described for aneurysm treatment *via* coiling (18). To the best of our knowledge, this is the first report of a direct percutaneous V2 puncture for MT of a BAO (2). We preferred roadmap guidance as we already had a catheter positioned in the proximal subclavian artery and were thus able to visualize both the carotid and the vertebral artery for guidance. The lateral approach offers more space for the operator and more degrees of freedom for the needle movement than the anterior approach, especially in the patients with short necks. Furthermore, the separated visibility of the VA from the CA when a lateral approach is chosen in comparison to ventral access argues in favor of this approach (17). As there is no dedicated material on the market for direct cervical punctures, we used a standard short femoral sheath in combination with a standard micropuncture kit. The puncture was straightforward as were the exchange maneuvers. Owing to the relative proximity of the sheath end and the embolus, wireless advancement of the aspiration catheter and subsequent direct aspiration maneuver were smooth and successful. Problems arose from the relatively small sheath that had been chosen as the thrombus was ultimately occluding the sheath. A larger sheath would have likely been advantageous in this respect. Yet, one has to weigh the benefits of a large sheath against the problems of puncture site closure. The possibility of vessel injuries, such as dissection, occlusion, distal embolism, arteriovenous fistula, and cervical compressive hematomas, as well as brachiocervical plexus nerve injury need to be critically considered, especially in a patient being on systemic thrombolytic, anticoagulation, or antiplatelet therapy. The authors Semeraro et al. and Blance et al. reported the use of a vascular closure system for DVP in their recently published case series to minimize the potential risk of cervical hematoma. In our case, 15 min of focal manual compression was efficient to achieve durable hemostasis (3, 19). A structured summary of the different DVP access techniques for MT in patients with acute BAO is given in Table 1. Alongside different DVP techniques, also surgical cutdowns have been proposed for direct VA access (20). In comparison, ultrasound- and/or roadmap-guided DVP of the V2 or V3 segment are less likely time-consuming and thus preferable. One could also consider leaving the sheath after the procedure and closing the arteriotomy surgically once the situation is less time-sensitive. Our presented technique is facilitated by the use of road mapping control which is able to localize precisely the target artery to be punctured. As most DVP procedures are likely for bail-out access in MT transradial or transfemoral diagnostic catheters are likely to be in a position to provide some sort of VA roadmap. Our report adds to the extremely limited direct VA access experience, describes a different technical nuance compared to similar approaches describes in the literature (2), and illustrates the procedure in detail, which might be of interest to physicians performing MT.

**Table 1:** Comparison of direct vertebral artery access techniques for mechanical thrombectomy in patients with acute basilary artery occlusion.

**Author**	**Date of publication**	**Access technique**	**Vertebral segment**	**Access according to anatomical landmark**	**Puncture site treatment**	**Complications**
Present case	-	Roadmap guided	V2	Posterolateral approach with needle introduced at the level of the process of C4 and C5	Manual compression	None
Elhorany et al. (2)	Jun 2020	Roadmap guided	V3	Posterolateral approach with needle introduced inferior to the tip of the mastoid process.	Manual compression	None
Semeraro et al. (3)	Jan 2021	Ultrasound-guided approach	V2	US probe placed oriented longitudinally to the V2, needle introduced between the transverse process of C4 and C5.	Vascular closure system (FemoSel, Terumo)	None
O'Reilly et al. (4)	Aug 2019	Ultrasound-guided approach	V3	US probe placed below the ipsilateral mastoid process, needle introduced proximal to the main collateral VA feeding branch	NA	None
Desai et al. (5)	March 2014	Ultrasound-guided approach	V3	US probe placed in the transverse orientation to the VA, needle introduced at the V3 segment inferior to the tip of the mastoid process.	NA	None

*the US, ultrasound; V2, segment 2 of the vertebral artery segment 2; V3, segment 3 of the vertebral artery*.

To conclude the DVP approach *via* roadmap-guided fluoroscopy may be a reasonable bail-out vascular access method for MT in patients with BAO in whom standard techniques remain futile. Prior road mapping of the targeted artery *via* femoral or radial approach may allow for straightforward fluoroscopy-guided cervical VA puncture and effective vessel recanalization in otherwise impossible cases. The size of the sheath and potential complications of this access must be considered and weighed against the natural history of the disease aimed to treat. Although our patient did not fare well in the end, it is fair to state that DVP did not attribute to this outcome.

## Take-Home Messages

- Direct percutaneous puncture (DVP) of the VA appears to be a valuable rescue access strategy for MT in patients with BAO when standard access techniques fail.
- The V2 segment of the VA can be directly punctured by a lateral cervical approach.
- Direct VA puncture (DVP) can be facilitated by roadmap guidance.
- The material commonly employed for transfemoral access can be successfully used in DVP.
- Safety aspects of this technique must be considered and weighed against the natural history of the disease aimed to treat.

## Data Availability Statement

The original contributions presented in the study are included in the article/supplementary material, further inquiries can be directed to the corresponding author.

## Ethics Statement

Ethical review and approval was not required for the study on human participants in accordance with the local legislation and institutional requirements. The patients/participants provided their written informed consent to participate in this study.

## Author Contributions

JN and ES: study design, acquisition of data, image processing, image analysis, data analysis, statistical analysis, and drafting the manuscript and revising it critically. GB: data analysis and drafting the manuscript and revising it critically. All authors contributed to the article and approved the submitted version.

## Funding

JN is grateful for being part of the BIH Charité – Digital Clinician Scientist Program funded by the Charité - Universitaetsmedizin Berlin, the Berlin Institute of Health, and the German Research Foundation (DFG, Deutsche Forschungsgemeinschaft).

## Conflict of Interest

The authors declare that the research was conducted in the absence of any commercial or financial relationships that could be construed as a potential conflict of interest.

## Publisher's Note

All claims expressed in this article are solely those of the authors and do not necessarily represent those of their affiliated organizations, or those of the publisher, the editors and the reviewers. Any product that may be evaluated in this article, or claim that may be made by its manufacturer, is not guaranteed or endorsed by the publisher.
